# Risk of Guillain-Barré syndrome after 2010–2011 influenza vaccination

**DOI:** 10.1007/s10654-013-9797-8

**Published:** 2013-03-31

**Authors:** Francesca Galeotti, Marco Massari, Roberto D’Alessandro, Ettore Beghi, Adriano Chiò, Giancarlo Logroscino, Graziella Filippini, Maria Donata Benedetti, Maura Pugliatti, Carmela Santuccio, Roberto Raschetti, The ITANG study group

**Affiliations:** 1 National Centre for Epidemiology, Surveillance and Health Promotion, National Institute of Health, Rome, Italy; 2 Neuroepidemiology Unit, I.R.C.C.S. Istituto delle Scienze Neurologiche, Bologna, Italy; 3 Laboratory of Neurological Disorders, “Mario Negri” Institute for Pharmacological Research, Milan, Italy; 4 Department of Neuroscience, University of Turin, Turin, Italy; 5 Department of Neurology and Psychiatry, University of Bari, Bari, Italy; 6 Neuroepidemiology Unit, I.R.C.C.S. Foundation, Carlo Besta Neurological Institute, Milan, Italy; 7 Department of Neurosciences, University Hospital, Verona, Italy; 8 Department of Clinical and Experimental Medicine, University of Sassari, Sassari, Italy; 9 Italian Medicine Agency (AIFA), Rome, Italy

**Keywords:** Influenza vaccination, Guillain-Barrè Syndrome, Case–control study, Self controlled case series

## Abstract

Influenza vaccination has been implicated in Guillain Barré Syndrome (GBS) although the evidence for this link is controversial. A case–control study was conducted between October 2010 and May 2011 in seven Italian Regions to explore the relation between influenza vaccination and GBS. The study included 176 GBS incident cases aged ≥18 years from 86 neurological centers. Controls were selected among patients admitted for acute conditions to the Emergency Department of the same hospital as cases. Each control was matched to a case by sex, age, Region and admission date. Two different analyses were conducted: a matched case–control analysis and a self-controlled case series analysis (SCCS). Case–control analysis included 140 cases matched to 308 controls. The adjusted matched odds ratio (OR) for GBS occurrence within 6 weeks after influenza vaccination was 3.8 (95 % CI: 1.3, 10.5). A much stronger association with gastrointestinal infections (OR = 23.8; 95 % CI 7.3, 77.6) and influenza-like illness or upper respiratory tract infections (OR = 11.5; 95 % CI 5.6, 23.5) was highlighted. The SCCS analysis included all 176 GBS cases. Influenza vaccination was associated with GBS, with a relative risk of 2.1 (95 % CI 1.1, 3.9). According to these results the attributable risk in adults ranges from two to five GBS cases per 1,000,000 vaccinations.

## Introduction

Guillain-Barré Syndrome (GBS) is an acute acquired immune-mediated polyradiculoneuropathy. It is an uncommon disease with crude incidence ranging from 0.81 to 1.89 cases per 100,000 person-years [1].

Despite medical treatment, GBS remains a severe condition: 3–10 % of patients die and 20 % are still unable to walk after 6 months [2]. The etiology of GBS is not yet completely understood, but it is preceded by an infectious disease in about two thirds of cases [2].

Evidence of a significantly increased incidence of GBS after swine influenza vaccination in the USA in 1976 led to a debate on the possible link between influenza vaccination and GBS [3]. The reported relative risk (RR) was 7.6 (95 % CI 6.7, 8.6) corresponding to about ten excess cases of GBS per million vaccinations.

Between 1978 and 2009 several studies were conducted yielding conflicting results [4–12]. Although the biological mechanisms responsible for the association between influenza vaccination and GBS remain unsettled [13], a study in mice [14] suggested that influenza vaccine antigens may induce cross-reactive anti-ganglioside antibodies eventually causing peripheral nerve damage.

During the influenza pandemic in 2009, the possible link between influenza vaccination and GBS drew special attention due to the rapid development and implementation of vaccines against pandemic influenza A/H1N1 virus (partially of swine origin as in 1976).

In Italy seasonal influenza vaccination is recommended, and is free of charge for adults aged ≥65 years and subjects with chronic diseases. Different types of inactivated vaccines (containing subtypes A/H1N1, A/H3N2, B) were available in Italy in 2010, mostly non-adjuvanted, one adjuvanted with MF59 and one containing virosomes.

In 2010 a prospective matched case–control study was launched in Italy to analyse the relation between exposure to seasonal influenza vaccination and subsequent onset of GBS. To complement the results of this study a self-controlled case series analysis was also planned to take into account potential biased selection of controls, recall bias and residual confounding.

## Materials and methods

### Study setting and population

Seven Italian Regions participated in the study: Lombardy, Piedmont, Valle d’Aosta, Veneto, Emilia Romagna, Puglia and Sardinia. The catchment area included ~29.5 million inhabitants, i.e., nearly half of the Italian population [15]. All the 152 neurological centers operating in the hospitals of the above Regions were contacted, 121 agreed to participate, of which 86 have contributed at least one case of GBS.

### Case definition

Consecutive subjects presenting with a clinical manifestation suggesting GBS or its variants between October 1, 2010 and May 15, 2011 (i.e. from the beginning to 6 weeks after the official end of the national vaccination campaign) were prospectively identified by the reporting neurologists and registered through a web-based data entry system. The level of diagnostic certainty of the cases was assessed in a uniform and objective way through the on-line verification tool (ABC-tool) as developed by the Brighton Collaboration [16]. All incident cases ≥18 years old fulfilling the case definition for GBS or its main variant, the Miller Fisher syndrome, and assigned to levels 1–3 according to the Brighton Collaboration definition, (where level 1 is the level of the highest diagnostic certainty) were included in the analyses. The date of onset of the first neurological symptom, as reported in clinical charts, was considered the case index date. Completeness of case reporting by the clinical centers was verified through regional hospital administrative discharge data (ICD-9 code 357.0) in four of the seven participating Regions (Lombardy, Piedmont, Valle d’Aosta, Emilia Romagna; more than 19 million inhabitants). Data were not available for the other Regions.

### Selection of controls

Controls were selected among patients admitted for acute conditions unrelated to chronic diseases (e.g. trauma) to the Emergency Department of the same hospital as the cases. Each control was individually matched to a case for admission date (i.e. the same date as the case or up to 30 days afterwards), sex, age (±5 years), and Region of residence.

### Data collection

Nine trained clinical research assistants visited the clinical centers to interview cases and controls and collect data from clinical charts. An ad-hoc report form was used to collect information related to suspected GBS diagnosis, influenza vaccination and other covariates i.e. exposure to drug treatments, pregnancy, influenza-like illness (ILI), upper respiratory tract infections (URI), gastrointestinal infections (GI) and other vaccinations in the previous 6 months, Epstein Barr virus past infection, past surgical interventions and chronic comorbidities such as malignancy, immunosuppression, autoimmune disorders. ILI, URI and GI events were defined as any reported episode lasting more than 24 h of fever >37.5 °C and malaise, fever with cough, nausea and vomiting or diarrhea, respectively. All the report forms were registered in the web-based system.

Information on 2010–2011 influenza vaccination (date and brand of vaccine) was verified by contacting patients’ general practitioners (GPs) by telephone. A neurologist (FG) closely verified and queried data quality.

### Time window at risk

The time window at risk was set at 6 weeks after vaccine administration, a generally accepted risk interval between GBS onset and exposure to an antigenic stimulus (e.g. infectious illness, vaccination), based on the biological plausibility for a causal relationship [17]. Sensitivity analyses were performed using two additional different definitions for the risk windows: 4 and 2 weeks after vaccine administration.

### Statistical analyses

Statistical analyses were conducted following two approaches: a matched case–control analysis and a self-controlled case series analysis.

#### Matched case–control analysis

Each case was individually matched to one up to four, if available, hospital controls. Cases and controls were defined as “exposed” when influenza vaccination occurred in the time window at risk before the index date, otherwise they were classified as “not exposed” (reference category).

Matched odds ratios and 95 % confidence intervals were calculated first by univariate conditional logistic analysis and subsequently by multivariate conditional logistic regression, after verifying the absence of multicollinearity. Factors associated with GBS by univariate analysis (*P* < 0.10) were considered eligible for inclusion in a multivariate model and retained in the model according to a forward stepwise procedure based on a likelihood-ratio test.

Analyses were performed with the software STATA version 11.2 (STATA Corp, College Station, TX, USA).

#### Self-controlled case series analysis

This analysis was based on the method developed by P. Farrington for Self-Controlled Case Series data (SCCS) [18, 19] using data only on cases. Originally designed to analyse the association between vaccinations and recurrent events, the method was adapted to outcomes, like GBS, where the occurrence of the event censors, curtails or otherwise affects post-event exposures (case series analysis for censored, perturbed or curtailed post-event exposures—SCCSadj) [20].

The method allows for the control of all permanent characteristics of patients in addition to seasonal variation in risk.

**Fig. 1:**
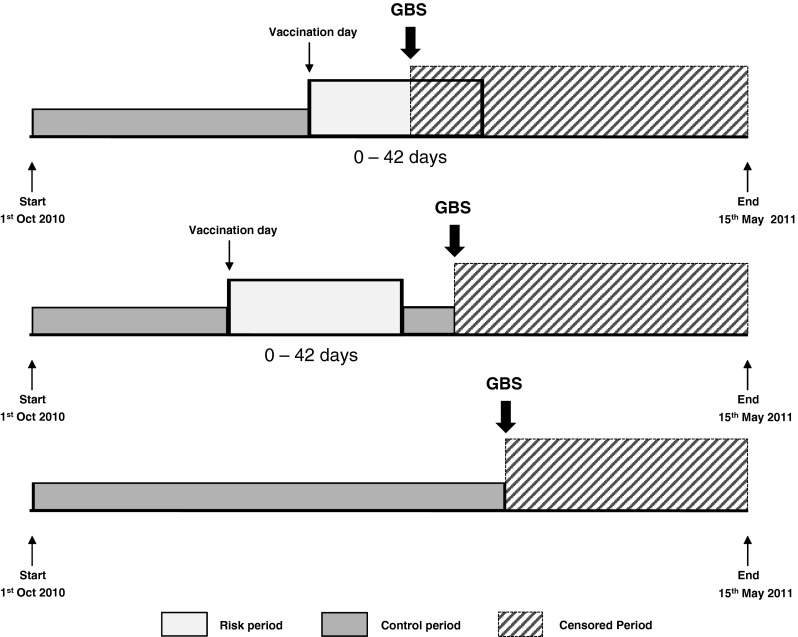
Diagram of the observation period for the self-controlled case-series (SCSS) method

All GBS cases (vaccinated and unvaccinated) recruited between October 1, 2010–May 15, 2011 were included in the analysis. The individual observation period for each case spanned from October 1, 2010 until onset of the first neurological symptom. The risk period was defined as the 6 weeks following vaccination. The remaining time was included as a control period representing the study baseline. A case was classified as exposed if GBS had onset during the risk period, and not exposed otherwise (Fig. 1).

Seasonal variation in GBS incidence was taken into account by dividing the individual observation period by calendar month. The incidence density of GBS during periods exposed and not exposed was compared. Relative Risk (RR) was computed by a conditional Poisson regression model providing an overall estimate of the effect of vaccination. Separate analyses were also conducted for ILI, URI and GI. The analyses were performed with R statistical package [21] where a specific routine for SCCS_adj_ was developed by Kuhner and Whitaker [22]. 95 % CIs were estimated applying the non-parametric bootstrap (10,000 replications) [23].

### Ethical aspects

The study protocol was approved by the Ethical Review Committees of the coordinating center (University of Bologna), the Italian National Institute of Health and of all participating clinical centers. All participants signed an informed consent form. Only anonymous datasets were shared for centralised analysis.

## Results

Between October 1, 2010 and May 15, 2011, 253 individuals with suspected GBS were observed in 86 adult neurology centers in the seven Italian Regions. Among these patients 12 (4.7 %) refused to participate, seven (2.8 %) were excluded due to a history of GBS and five (2 %) for whom the GBS diagnosis was not confirmed (the final diagnoses were: paraneoplastic neuropathy; chronic inflammatory demyelinating polyradiculoneuropathy; lymphoma infiltration of spinal nerve roots; paraneoplastic autoimmune encephalitis; Lyme neuroborreliosis).

**Fig. 2:**
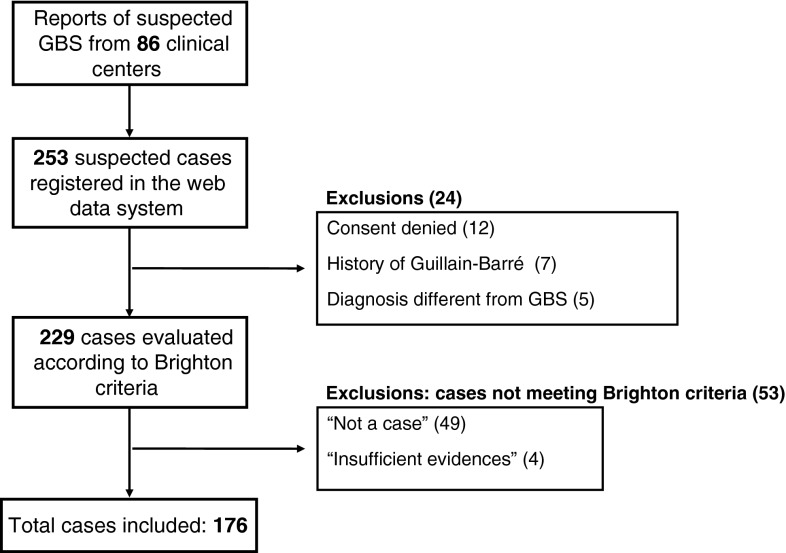
Recruitment of participants in the study. The reasons for exclusion from the Brighton criteria were: alternative explanation of symptoms (11 cases), course not monophasic (11), nadir of symptoms not reached between 12 h and 28 days after symptoms onset (5), absence of bilateral limbs weakness (18), conservation of tendon stretching reflexes (4); missing data (4 patients)

According to the Brighton Classification, 176 patients met level 1–3 criteria for GBS or Miller Fisher syndrome and were included in the study (Fig. 2). The 53 GBS cases excluded because they did not fulfil Brighton criteria for case definition did not differ from the included cases in frequency of seasonal influenza vaccination and in frequency of occurrence of other covariates.

The median age of included cases was 63 years (range 19–96). More than half of patients scored ≥4 according to the GBS disability scale [17]. The main clinical features of the GBS cases are summarised in Table 1.

**Table 1:** Clinical features of Guillain-Barré syndrome cases included in the study (n = 176)

	No	%	Mean (SD)
Mean age at hospital admission			60.1 (17.5)
≥65 years	84	47.7	
Male	100	56.8	
Clinical symptom at onset			
Motor deficit	77	43.7	
Sensory impairment	51	29.0	
Sensory-motor deficit	38	21.6	
Other	8	4.6	
Unknown	2	1.1	
Brighton collaboration case classification^a^			
Guillain–Barré syndrome			
Level 1	115	65.3	
Level 2	53	30.1	
Level 3	5	2.8	
Miller Fisher syndrome			
Level 1	1	0.6	
Level 2	2	1.2	
Level 3	0	0	
Disability score (%)			
0–1 (minimal or no deficit)	15	8.5	
2 (able to walk 10 m unassisted, but unable to run)	20	11.4	
3 (able to walk 10 m over open space, with help)	40	22.7	
4 (bedridden or chair-bound)	84	47.7	
5–6 (needs ventilator at least for part of the day or deceased)	17	9.7	

^a^Clinical case definitions according to the Brighton Collaboration Criteria: Guillain–Barré Syndrome or Miller Fisher syndrome (MFS): Level 1 of diagnostic certainty: clinical, electrophysiological and cerebrospinal fluid (CSF) findings consistent with GBS (or MFS), in the absence of an identified alternative diagnosis. Level 2 of diagnostic certainty: clinical and either electrophysiological or CSF findings consistent with GBS (or MFS), in the absence of an identified alternative diagnosis. Level 3 of diagnostic certainty: only clinical findings consistent with GBS (or MFS), in the absence of an identified alternative diagnosis

The 53 centers from the four Regions in which completeness of case reporting was verified against hospital administrative discharge data contributed to 68.7 % of the cases included. Case reporting was complete in 37 centers while 21 cases with a clinically confirmed diagnosis and with no evident reason for study exclusion, coming from 16 clinical centers, were not registered through the web-based data entry system. These cases showed no meaningful difference in the proportion of influenza vaccination or other covariates compared with included cases.

### Case–control analysis

Of the 176 GBS cases, 140 (79.5 %) were individually matched to 308 controls. Thirty-six cases were excluded because their recruited controls did not meet one or more of the required matching criteria, or the diagnoses of recruited controls were not related to an acute condition.

Among matched controls the most frequent diagnoses were acute cerebrovascular episodes (31.8 %) and trauma (24.4 %). Other acute conditions included: acute cardiac disorders (10.4 %), first episodes of loss of consciousness and acute headache (10.4 %), and vertigo (4.2 %). The characteristics of cases and controls are reported in Table 2.

**Table 2:** Characteristics of cases and controls included in the study

	Cases (n = 140)			Controls (n = 308)			
	No	%	Mean (SD)	No	%	Mean (SD)	*P* value
Mean age			60.6 (17.8)			62.1 (17.1)	0.40
≥65 years	70	50.0		169	54.9		0.36
Male	80	57.1		179	58.1		0.92
Vaccinations in 6 weeks before index date							
Seasonal influenza vaccination 2010–2011	20	14.3		27	8.8		0.10
Other vaccinations	1	0.7		1	0.3		0.53
Infections in 6 weeks before index date (%)							
Influenza-like illness (ILI)	40	28.6		12	3.9		<0.001
Upper respiratory tract infection (URI)	37	26.4		25	8.1		<0.001
Gastrointestinal infection (GI)	30	21.4		8	2.6		<0.001
Chronic comorbidity							
Malignancy	15	10.7		34	11.0		0.99
Immunocompromised	9	6.4		4	1.3		0.005
Autoimmune disorders	11	7.9		11	3.6		0.06
Epstein-Barr virus	7	5.0		7	2.3		0.15
Other conditions in 6 months before index date							
Pregnancy	0	0.0		2	0.6		0.99
Surgery	8	5.7		14	4.5		0.64
Influenza-like illness (ILI)	45	32.1		31	10.1		<0.001
Upper respiratory tract infection (URI)	42	30.0		45	14.6		<0.001
Gastrointestinal infection (GI)	33	23.6		17	5.5		<0.001
Drug use: n (Mean)	403	2.9		777	2.5		0.18
Patients with >5 drugs	19	13.6		42	13.6		0.99
Vaccination							
Seasonal influenza vaccination 2010–2011	45	32.1		85	27.6		0.37
Seasonal influenza vaccination >6 weeks	25	17.9		58	18.8		0.90
Other vaccinations	2	1.4		5	1.6		0.99

The proportion of missing data for the different factors investigated was in general very low (1.4 % among cases and 1.6 % among controls). Only for the possible Epstein-Barr virus infection the proportion of missing data was high but not differentially between cases and controls (36.0 and 36.4 % respectively).

In about 67.1 % of cases GBS was preceded by an infectious disease (ILI or URI or GI).

Drug use in the 6 weeks preceding the index date was similar for cases and controls (mean number of reported drugs: 2.9 and 2.5 respectively; more than five drugs 13.6 % both in cases and controls).

The proportion of seasonal influenza vaccinations outside the time window at risk was nearly the same for cases and for controls (17.9 vs. 18.8 % respectively). Vaccinations were provided mainly by general practitioners (82.8 %). After up to four calls, 50 % of GPs was contacted and interviewed to validate information about influenza vaccination. Information provided by patients (68.6 % of cases and 42.3 % of controls) was always confirmed by the general practitioners.

No relationship between vaccination status and severity of GBS (measured through the disability scale) was detected (Pearson Chi square = 8.2, *P* = 0.223).

The results of univariate conditional regression analysis are reported in Table 3.

**Table 3:** Univariate (^m^OR) conditional regression analysis

	^m^OR	95 % CI	*P*
Vaccinations in 6 weeks before index date			
Seasonal influenza vaccination 2010–2011	3.9	1.6–9.9	0.004
Other vaccinations	3.5	0.2–55.8	0.38
Infections in 6 weeks before index date			
Influenza-like illness (ILI)	8.7	4.2–18.3	<0.001
Upper respiratory tract infection (URI)	4.1	2.2–7.7	<0.001
Gastrointestinal infection (GI)	12.3	4.7–32.2	<0.001
Chronic comorbidity			
Malignancy	1.2	0.6–2.4	0.65
Immunocompromised	6.1	1.6–23.3	0.008
Autoimmune disorders	2.7	1.1–7.0	0.035
Epstein-Barr virus	2.2	0.7–6.4	0.15
Other conditions in 6 months before index date			
Surgery	1.0	0.4–2.5	0.98
Influenza-like illness (ILI)	4.1	2.3–7.1	<0.001
Upper respiratory tract infection (URI)	2.7	1.6–4.7	<0.001
Gastrointestinal infection (GI)	5.9	2.9–11.8	<0.001
Vaccination			
Seasonal influenza vaccination 2010–2011	1.6	0.9–2.7	0.12
Seasonal influenza vaccination >6 weeks	0.9	0.4–1.6	0.64
Other vaccinations	1.0	0.2–5.6	0.98

**Fig. 3:**
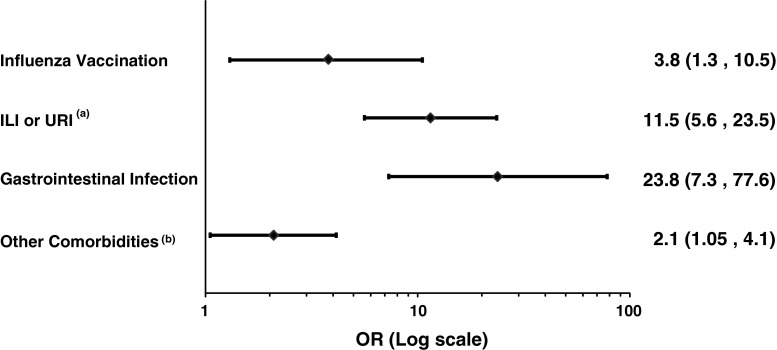
Forest plot showing the results of the final conditional regression analysis. ^a ^ILI = Influenza-like illness; URI = Upper respiratory tract infections, ^b^ Any occurrence of chronic comorbidities among: malignancies, immunocompromised states (e.g. transplantation, use of immunosuppressants, HIV infection), autoimmune disorders (e.g. thyroiditis, diabetes type I, rheumatoid arthritis, vasculitis) and past surgical intervention

In the multivariate final model we defined two composite variables: the first considered any occurrence of ILI or URI (as the two may be misclassified) and the second any occurrence of chronic comorbidities (i.e. malignancies, immune-compromised status, autoimmune disorders) or surgical interventions. The results of the final conditional regression analysis are reported in Fig. 3 as a forest plot.

Exposure to influenza vaccine was associated to GBS, with a matched adjusted odds ratio of 3.8 (95 % CI 1.3, 10.5). The matched adjusted odds ratio for GI was 23.8 (95 % CI 7.3, 77.6), for URI or ILI was 11.5 (95 % CI 5.6, 23.5). Other comorbidities were also associated with GBS (matched adjusted odds ratio: 2.1; 95 % CI 1.05, 4.1). A sensitivity analysis carried out with only cases with highest level of diagnostic certainty according to the Brighton Classification (level 1) showed similar results. The matched adjusted odds ratio for influenza vaccine was 3.5 (95 % CI 1.1, 11.3).

Within the time window at risk, the median interval between vaccination and the onset of the first neurological symptom was 14 days (range 9–39).

Analyses considering the 4 and 2 weeks risk periods confirmed the association between influenza vaccine and GBS: the matched adjusted odds ratio in the 4 weeks period was 3.5 (95 % CI 1.1, 11.1) and in the 2 weeks period 4.7 (95 % CI 1.3, 17.8).

Finally, to face with a possible selection bias among the cases, we restricted the analysis to the cases coming from centers with complete reporting as verified through hospital administrative discharge data (37 centers). The matched adjusted odds ratio for influenza vaccine was 4.9 (95 % CI: 1.3, 19.2) which overlaps with the result from including all the cases.

### Case-series analysis

The SCCS included all the 176 GBS cases. Among these, 24 cases occurred within 42 days from receiving influenza vaccine, and 29 had the onset of GBS out the at risk time window.

Adopting the SCCS_adj_ analysis, the RR was 2.1 for seasonal influenza vaccination (95 % CI 1.1, 3.9), 15.6 for URI or ILI (95 % CI 7.1, 41.9) and 41.4 for GI (95 % CI 9.0, 90.8). When the analysis was restricted to the 140 cases considered for the case–control analysis the RR for influenza vaccination was 2.0 (95 % CI 1.01, 4.0).

## Discussion

Ours is the first study conducted during the 2010–2011 vaccination campaign and demonstrates a statistically significant association between seasonal influenza vaccines (containing subtypes A/H1N1, A/H3N2, B) and occurrence of GBS.

Selection biases are unlikely to explain the results we observed. For the case–control analysis, selection bias would occur if the controls were not representative for the exposure of interest in the source population or if the cases had been selected according to exposure status.

On the basis of the official data from the Italian Ministry of Health, vaccination coverage in the adult general population in the participating Regions was 18.1 % [24]. Considering the 308 controls included in the case–control analysis, and after applying a direct standardization method, the figure was very similar (17.3 %). Using an “indirect” standardization procedure the expected number of vaccinees among the 308 controls was 100 and the observed vaccinees were 91 (i.e. Indirectly Standardized Rate, ISR = 0.91).

The quality of data in a case–control study is determined to a large extent by the patient’s ability to recall past exposure(s) accurately. The timeliness of gathering information minimizes this kind of bias. In our study information on the main exposures (infections and vaccinations) was collected after a short interval in both cases and controls, thereby minimizing the possibility of recall bias. Moreover, when the vaccine use was verified through general practitioners they always confirmed the information provided by the subjects in study, both cases and controls. Despite this reassuring result, the relatively low percentage for which the exposure could be verified by a GP remains a potential limitation of our study.

Biases could also have occurred during interviewing because interviewers were unblinded to diagnoses and they could have been more accurate when interviewing cases than when interviewing controls, tending assertively to find exposure. However, the frequency of vaccination outside the time window at risk broadly overlapped between cases and controls, making an interviewer bias unlikely. Finally, the proportion of missing data was in general very low.

The case-series analysis (based on cases only) also confirms the association between this vaccination and GBS (RR = 2.1). In this approach each case acted as his/her own control, inherently taking into account confounding factors that did not vary with time over the observation period. Moreover, as no separate controls are needed potential selection and information bias are reduced. The width of the confidence intervals in the two types of analysis of our study is rather large, so that the differences in the point estimate may be due to random variability.

A selection bias among the cases may have occurred if cases exposed to vaccination, in the time window at risk, were more likely to be included in our study than non exposed cases. However, in the Regions where completeness of case identification was verified through hospital administrative discharge data, a comparison between the cases included in the study from the clinical centers with some missing cases and those from the clinical centers where all the admitted GBS patients were included, did not disclose a meaningful difference in the proportion of exposed cases. Also the multivariate conditional analysis restricted to clinical centers where all the patients were included confirms that a selection bias is unlikely to explain the association between influenza vaccination and GBS.

Moreover, an objective algorithm (Automatic Brighton Criteria) was applied for case inclusion in the analysis, without knowledge of exposure status. In addition, the clinical characteristics of cases included in the study (e.g. frequency of antecedent infections, male/female ratio) overlap broadly with those described in the literature (2).

Main strengths of this study are that it is large and statically powerful. Moreover the results of the case–control study were confirmed by the self controlled case series analysis, which is less prone to problems of confounding.

Eight studies were conducted during the 2009–2010 influenza vaccine campaign with monovalent A/H1N1 vaccine.

In the United States, data from a population-based surveillance program conducted by the Centers for Disease Control and Prevention (CDC), comparing A/H1N1 vaccinated to unvaccinated persons [25] showed an age-adjusted rate ratio of 1.8 (95 % CI 1.1, 2.6). A further self-controlled analysis of the data from the CDC surveillance program [26], showed a relative risk of 2.1 (95 % CI 1.2, 3.5). Other three studies [27–29] were carried out in USA and recently published. These studies found a small increase of approximately 1 case of GBS per million vaccines above the baseline rate. Another study [30] conducted in five European countries found that the receipt of pandemic influenza vaccine (A/H1N1) was not associated with an increased risk of GBS, but data from UK highlighted an association between GBS and exposure to seasonal influenza vaccination (OR = 5.1; 95 % CI 1.4, 18.6). A case–control study in France [31] did not support the association between GBS occurrence within 6 weeks after seasonal vaccination (OR = 1.3; 95 % CI 0.4, 4.1) and A/H1N1 vaccination (OR = 0.9; 95 % CI 0.1, 7.6). But this study had limited statistical power to detect an association of the magnitude of a few cases per 1 million doses.

Finally a population-based cohort study was conducted in Quebec, Canada [32]. In this study the 2009 influenza A/H1N1 vaccine was associated with a small but significant risk of GBS (about 2 cases per 1 million doses in the 4 weeks following vaccine administration).

Overall the results of our study do not modify the risk–benefit profile of seasonal influenza vaccination. According to our results the attributable risk in adults ranges from two to five GBS cases per 1,000,000 vaccinations. As with all vaccine-related events, there is the need to balance the potential risk of vaccine-related adverse events against vaccine effectiveness.

Influenza infection is a major public health problem. Estimates show that seasonal influenza causes 8,000 excess deaths in Italy annually, 1,000 due to pneumonia and influenza and 7,000 due to all causes [33]. Vaccination remains the most important counter-measure for preventing influenza virus infection and its complications [34, 35].

## Appendix: The ITAlian Network for the study of GBS (ITANG)

### Clinical research assistants

Sara Marconi (Department of Neurological Sciences, Neuroepidemiology Unit, University of Bologna, Italy), Giorgia Giussani (Laboratory of Neurological Disorders, Department of Neuroscience, “Mario Negri” Institute for Pharmacological Research Milan, Italy), Enrica Bersano and Umberto Manera (Department of Neuroscience, University of Turin, Italy), Stefano Zoccolella and Antonio Leo (Department of Neurology and Psychiatry, University of Bari, Italy), Roberta Barki (Neuroepidemiology Unit, I.R.C.C.S. Foundation Carlo Besta Neurological Institute, Milan, Italy), Marco Turatti (Department of Neurological, Neuropsychological, Morphological and Movement Sciences, University of Verona, Italy), Stefania Leoni (Department of Clinical and Experimental Medicine, University of Sassari, Italy).

### Steering committee of the study

Ettore Beghi (Laboratory of Neurological Disorders, “Mario Negri” Institute for Pharmacological Research, Milan, Italy), Maria Donata Benedetti (Department of Neurosciences, University Hospital, Verona, Italy), Andrea Calvo and Adriano Chiò (Department of Neuroscience, University of Turin, Italy), Roberto D’Alessandro (*Chair*, Neuroepidemiology Unit, I.R.C.C.S. Istituto delle Scienze Neurologiche, Bologna, Italy), Fernanda Ferrazin (Italian Medicine Agency, AIFA), Graziella Filippini (Neuroepidemiology Unit, I.R.C.C.S. Foundation Carlo Besta Neurological Institute, Milan, Italy), Giancarlo Logroscino (Department of Neurology and Psychiatry, University of Bari, Italy), Giampiero Mazzaglia (Health Search, Italian College of General Practitioners, Florence, Italy), Maura Pugliatti (Department of Clinical and Experimental Medicine, University of Sassari, Italy), Roberto Raschetti (National Centre for Epidemiology, Surveillance and Health Promotion, National Institute of Health, Rome, Italy), Carmela Santuccio, Loriana Tartaglia and Francesco Trotta (Italian Medicine Agency, AIFA).

### Clinical centers and co-investigators

#### E. Romagna

Roberto MICHELUCCI, Patrizia RIGUZZI: **UO Neurologia Ospedale Bellaria-IRCCS Istituto delle Scienze Neurologiche, Bologna, Italy;**

Tommaso SACQUEGNA, Anna Maria BORGHI: **UO neurologia Ospedale Maggiore-IRCCS Istituto delle Scienze Neurologiche, Bologna, Italy;**

Vittoria MUSSUTO, Patrizia DE MASSIS: **Struttura Semplice Dipartimentale Di Neurologia—Ospedale Santa Maria Della Scaletta, Imola**;

Fabio CIRIGNOTTA, Rita Rinaldi: **U.O. Neurologia—Policlinico Sant’Orsola-Malpighi, Bologna**;

Walter NERI, Carlo GUIDI: **U.O. Neurologia—Ospedale Di Forlì—Azienda USL, Forlì**;

Maria Rosaria TOLA: **U.O. Neurologia—Azienda Ospedaliero—Universitaria Arcispedale Sant’Anna, Ferrara**;

Enrico GRANIERI, Valentina SIMIONI: **U.O. Neurologia—Università Di Ferrara**;

Paolo NICHELLI, Jessica MANDRIOLI: **Clinica Neurologica—Università di Modena e Reggio Emilia—Nuovo Ospedale C. Sant’Agostino e Estense**;

Donata GUIDETTI, Emilio TERLIZZI: **U.O. Neurologia—Ospedale Guglielmo Da Saliceto—Azienda USL, Piacenza**;

Mario Giovanni TERZANO, Giovanni PAVESI: **Dipartimento di Neuroscienze Università Degli Studi Di Parma**;

Fabrizio RASI, Claudio CALLEGARINI: **U.O. Neurologia—Ospedale Ravenna—Azienda USL**;

Norina MARCELLO, Massimo BONDAVALLI: **U.O. Neurologia—Azienda Ospedaliera di Reggio Emilia—Arcispedale S. Maria Nuova**;

Alessandro RAVASIO, Marco CURRÒ DOSSI: **U.O. Neurologia—Ospedale di Rimini—Azienda USL, Rimini**;

Annamaria MAURO, Chiara MINARDI: **U.O. Neurologia—Ospedale Bufalini—Azienda USL di Cesena;**

Gabriele GRECO, Stefano AMIDEI: **U.O. Neurologia -Ospedale di Carpi—Azienda USL di Modena;**

Enrico MONTANARI, Doriana MEDICI: **U.O Neurologia—Ospedale di Fidenza-San Secondo—AUSL Parma**;

#### Lombardy

Tommaso RICCARDI, Elisabetta D’ADDA: **Azienda Ospedaliera Ospedale Maggiore di Crema—U.O. Neurologia**;

Luigi BETTONI, Luciano ABRUZZI: **Azienda Ospedaliera di Cremona, Ospedale di Cremona—U.O. Neurologia-Stroke Unit**;

Elio AGOSTONI, Francesco BASSO: **Azienda Ospedaliera di Lecco, Presidio Ospedaliero “A. Manzoni” di Lecco—U.O. Neurologia-Stroke Unit**;

Andrea MAGNONI: **Azienda Ospedaliera di Lecco, Presidio Ospedaliero “S.L.Mandic” di Merate—U.O. Neurologia-Stroke Unit**;

Vittorio CRESPI, Maria REPACI: **Azienda Ospedaliera di Desio e Vimercate, Presidio di Vimercate—U.O. Neurologia**;

Antonella CHELDI, Mariagrazia BELLOTTI: **Azienda Ospedaliera di Desio e Vimercate, Presidio di Desio—U.O. Neurologia**;

Carlo FERRARESE, Natale Augusto CURTÒ: **Azienda Ospedaliera San Gerardo—Clinica Neurologica, Monza**;

Giovanni MEOLA, Giuseppe ROTONDO: **IRCCS Policlinico San Donato—U.O. Neurologia**;

Eduardo NOBILE-ORAZIO, Fabrizia TERENGHI: **Istituto Clinico Humanitas—U.O. Neurologia II, Rozzano**;

Franco SASANELLI, Alessio GALBUSSERA: **Azienda Ospedaliera Ospedale di Circolo di Melegnano, Presidio di Vizzolo Predabissi—U.O. Neurologia**;

Marco MATTIOLI, Marco TIRITICCO**Azienda Ospedaliera “Guido Salvini” U.S.C. Neurologia, Garbagnate Milanese**;

Caterina NASCIMBENE, Alessandra VANOTTI: **Azienda Ospedaliera “Ospedale Luigi Sacco”—U.O. Neurologia, Milano**;

Stefano JANN, Luisa DE TONI FRANCESCHINI: **Azienda Ospedaliera “Ospedale Niguarda Cà Granda”—Dipartimento di Neuroscienze, Neurologia e Stroke Unit, Milano**;

Vincenzo SILANI, Nicola TICOZZI: **I.R.C.C.S. Istituto Auxologico Italiano San Luca—U.O. Neurologia, Milano**;

Giancarlo COMI, Raffaella FAZIO: **Fondazione San Raffaele del Monte Tabor- U.O. Neurologia, Neurofisiologia Clinica e Neuroriabilitazione, Milano**;

Patrizia PERRONE, Andrea GIORGETTI: **Azienda Ospedaliera “Ospedale di Legnano”, Presidio di Legnano—U.O. Neurologia**;

Alessandro ROMORINI: **Azienda Ospedaliera “Ospedale di Legnano”, Presidio di Magenta—Servizio di Neurologia**;

Pietro BASSI, Domenico SANTORO: **Ospedale San Giuseppe F.B.F- U.O. Neurologia, Milano**;

Nereo BRESOLIN: **Fondazione IRCCS Cà Granda, Ospedale Maggiore Policlinico –U.O. Neurologia**;

Simone TONIETTI, Massimo SUARDELLI: **Azienda Ospedaliera “Ospedale San Carlo Borromeo”—U.O.C. Neurologia e Stroke Unit, Milano**;

Giacomo BEZZI, Daria BALDINI: **Azienda Ospedaliera della Valtellina e della Valchiavenna, Ospedale Civile Di Sondrio e Ospedale Di Sondalo- U.O. Neurologia**;

Angelo Maurizio CLERICI, Giuseppina CAFASSO: **Ospedale Di Circolo E Fondazione Macchi Varese—U.O. Neurologia e Stroke**;

Daniele PORAZZI, Isidoro LA SPINA: **Azienda Ospedaliera Ospedale di Circolo di Busto Arsizio, Presidio di Busto Arsizio—U.O. Neurologia**;

Giampiero GRAMPA: **Azienda Ospedaliera Ospedale di Circolo di Busto Arsizio, Presidio di Saronno—U.O. Neurologia**;

Davide ZARCONE, Michele PERINI: **Azienda Ospedaliera “S.Antonio Abate di Gallarate”—U.O. Neurologia;**

Marco POLONI, Emanuela AGAZZI**: Ospedale Riuniti di Bergamo**;

Massimiliano FILOSTO, Alessandro PADOVANI: **Azienda Ospedaliera Spedali Civili, Brescia**;

Renato BESANA: **Ospedale M. Mellini, Chiari**;

Edoardo DONATI, Eugenio MAGNI: **Fondazione Poliambulanza Brescia**;

Marco ARNABOLDI, Vincenzo BELCASTRO: **Ospedale Sant’Anna, Como**;

Mario GUIDOTTI, Raffaella CLERICI: **Ospedale Valduce, Como**;

Maurizio RIVA, Eugenio VITELLI: **Azienda Ospedaliera della Provincia di Lodi**;

Paolo PREVIDI **Mantova**;

Giuseppe MICIELI, Elisa CANDELORO: **Fondazione Irccs Ist. Neurologico C.Mondino, Pavia**;

Carlo DALLOCCHIO, Carla ARBASINO: **Azienda Ospedaliera di Pavia- Stabilimento di Voghera**;

#### Piedmont

Andrea CALVO: **Scdu Neurologia 4—AOU S. Giovanni Battista, Torino**;

Cristina MOGLIA: **Scdu Neurologia 4—AOU S. Giovanni Battista, Torino**;

Marco VERCELLINO: **Scdu Neurologia 3—AOU S. Giovanni Battista, Torino**;

Bruno FERRERO: **Scdu Neurologia I—AOU S. Luigi Gonzaga, Orbassano**;

Pietro PIGNATTA, Enrico ODDENINO: **Ospedale Gradenigo, Torino**;

Daniele IMPERIALE: **Osp. Maria Vittoria, Torino**;

Daniela LEOTTA: **Osp. Martini, Torino**;

Maurizio GIONCO: **Osp. Mauriziano, Torino**;

Carlo RAVETTI, Roberto CAVALLO: **Osp. San Giovanni Bosco, Torino**;

Nicoletta Di Vito: **Osp. Cardinal Massaia ASL 19 Asti**;

Claudio GEDA: **Osp di Ivrea**;

Simona BORTOLOTTO: **ASL TO5—Moncalieri, Chieri, Carmagnola, Nichelino**;

Emilio LUDA DI CORTEMIGLIA, Giovanna LIOTTA: **Osp.di Rivoli**;

Emilio URSINO, Mario PALERMO: **ASO SS. Antonio e Biagio e Cesare Arrigo di Alessandria**;

Luca AMBROGIO, Piero MEINERI: **ASO Santa Croce e Carle di Cuneo**;

Michele DOTTA: **Alba Bra A.S.L.18 Osp. San Lazzaro**;

Paolo GHIGLIONE, Joseph MAISTRELLI: **Osp. Santissima Annuinziata di Savigliano**;

Susana ONORATO: **Osp. Borgomanero**;

Maurizio LEONE: **AOU Maggiore di Novara**;

Diego Maria PAPURELLO: **Presidi Ospedalieri Riuniti di Cirié**;

Angelo VILLANI, Roberto CONTI: **Osp. San Biagio di Domodossola**;

Franco COPPO, Fabiana TESSER: **Presidio Osp. Sant’Andrea di Vercelli**;

Lucia Testa: **Osp. Santo Spirito—Casale Monferrato;**

Silvia Isabella Cattaneo: **Osp. San Giacomo—Novi Ligure;**

Maria Teresa Penza: **Osp. S.S. Antonio e Margherita—Tortona;**

Graziano Gusmaroli, Katia Savio: **Osp. Degli Infermi—Biella;**

Franco Perla: **Osp. Poveri Infermi—Ceva Mondovì;**

Fabrizio Pisano: **Fondazione Salvatore Maugeri, IRCSS, Istituto Scientifico di Veruno;**

Fabio Poglio: **Osp. Agnelli di Pinerolo;**

Luca Pradotto: **Istituto Auxologico Italiano, IRCSS—Osp. San Giuseppe—Piancavallo;**

#### Valle d’Aosta

Edo Fausto BOTTACCHI: **SC Neurologia, Ospedale di Aosta**;

#### Puglia

Paolo LIVREA: **Clinica Neurologica Amaducci—Universita’ Degli Studi Di Bari**;

Francesco FEDERICO: **Clinica Neurologica Fm Puca Policlinico Di Bari**;

Maria TROJANO: **U.O. Neurofisiopatologia Policlinico Bari**;

Vito COVELLI: **UO Neurologia Ospedaliera Policlinico Di Bari**;

Bruno MAGGIO: **UC Di Neurologia Ospedale San Giacomo, Bari**;

Vito SANTAMATO: **Divisione Di Neurologia Azienda Ospedaliera “Di Venere”, Bari**;

Bruno PASSARELLA: **U.O. Di Neurologia—Ospedale “A. Perrino”, Brindisi**;

Giovanni ZIMATORE: **Div. Neurologia Ospedale “Mons. Raffele Dimiccoli”, Barletta**;

Giorgio TRIANNI: **Reparto Di Neurologia—Ospedale Vito Fazzi, Lecce**;

Gerardo CIARDO: **U.O. Neurologia Azienda Ospedaliera “Cardinale G. Panico”, Tricase**;

Salvatore INTERNO’: **Divisione di Neurologia—Ospedale Civile, Taranto**;

#### Veneto

Corrado MARCHINI; Sandro ZAMBITO MARSALA: **Reparto di Neurologia—Ospedale Civile San Martino—USL 1 Belluno**;

Giorgio MICHIELI, Carlo BORSATO: **UOC di Neurologia Ospedale di Piove di Sacco—Azienda ULS 16**;

Giuseppe DIDONÈ, Emma FRASSON: **Divisione di Neurologia—Ospedale di Cittadella**;

Alberto POLO: **Divisione di Neurologia—Ospedale di Monselice**;

Vincenza ARGENTIERO, Vincenzo ROMEO: **Clinica Neurologica II—Ospedale S. Antonio, Padova**;

Novenia PERLOTTO: **Clinica Neurologica I—Ospedale Civile, Padova**;

Roberto L’ERARIO: **Divisione di Neurologia—Ospedale Civile, Rovigo**;

Gianfranco MICAGLIO, Tiziana ROSSO: **Divisione di Neurologia U.S.L. 8, Castelfranco Veneto**;

Sandro BRUNO: **Divisione di Neurologia—Ospedale di Conegliano;**

Bruno GIOMETTO, Mirco SERENA: **Divisione di Neurologia—Ospedale Regionale Ca’ Foncello, Treviso**;

Rocco QUATRALE, Ernesto GASTALDO: **Divisione di Neurologia—Ospedale Umberto I, Mestre**;

Francesco PERINI, Michele DILEONE: **Divisione di Neurologia—Ospedale S. Bortolo, Vicenza**;

Alessandro CASANO, Lauretta SILVESTRI: **Divisione di Neurologia—Ospedale di Arzignano**;

Alessandro BURLINA, Virginia MUNERATI: **Divisione di Neurologia—Ospedale Nuovo, Bassano del Grappa**;

Sebastiano D’Anna: **Presidio Ospedaliero di Portogruaro;**

Flavio SANSON, Valeria SARTORI: **Divisione di Neurologia, Thiene**;

Giuseppe MORETTO, Giovanna SQUINTANI: **UO di Neurologia—Ospedale Civile Maggiore di Borgo Trento, Verona**;

Salvatore MONACO, Maria Donata BENEDETTI: **UO di Neurologia—Policlinico G. Rossi, Verona**;

Simone FUSINA: **Presidio Ospedaliero S. Bonifacio;**

Claudio BIANCONI, Fabiana PIMAZZONI: **Divisione di Neurologia—Istituto Don Calabria, Negrar**;

Angela BONOMETTI: **Divisione di Neurologia—Ospedale Mater Salutis, Legnago**;

Giampietro ZANETTE, Marco TURATTI: **Reparto di Neurologia—Casa di Cura Pederzoli, Peschiera del Garda**;

#### Sardinia

Stefania LEONI: **Dip. di Medicina Clinica e Sperimentale, Università degli Studi di Sassari**;

Giuseppe MURA: **Servizio di Neurologia—Ospedale San Giovanni Di Dio—ASL 2, Olbia;**

Anna TICCA: **Div. di Neurologia—Ospedale di Nuoro—ASL 3, Nuoro**;

Piernicola MARCHI: **Dip. di Scienze Cardiovascolari e Neurologiche—Sez. Neurologia—AOU Cagliari**;

Davide MANCA: **S.C. Neurologia e Stroke Unit—AO Brotzu, Cagliari.**
